# Supplementary material for: Risk of revision arthroplasty surgery after exposure to physically demanding occupational or leisure activities: A systematic review
Source: PLoS One. 2022 Feb 28;17(2):e0264487. doi: 10.1371/journal.pone.0264487 (PMC8884506; doi:10.1371/journal.pone.0264487)
Supplement: S1 File — (DOCX) [file pone.0264487.s001.docx]

**S1 File. Combination of MeSH terms and keywords used in the search**

**Embase** 1974 to 2021 July 07

|  | **Query** | **Results from 8 Jul 2021** |
| --- | --- | --- |
| 1 | (total knee replacement$ or knee replacement$ or knee arthroplast$ or gonarthroplasty or TKA or knee prosthesis).mp. | 59,019 |
| 2 | (hip prosthesis or hip replacement$ or total hip replacement$ or total hip arthroplast$ or THA).mp. | 63,057 |
| 3 | (long term or long-term or longterm).mp. | 1,282,938 |
| 4 | risk factor.mp. | 1,268,498 |
| 5 | exp total knee arthroplasty/ or exp knee replacement/ or exp knee arthroplasty/ or exp knee prosthesis/ | 39,875 |
| 6 | exp return to work/ | 7,385 |
| 7 | exp employment/ or exp employment status/ or exp manual labor/ or employ$.tw. or exp work/ or exp work capacity/ or work activit$.tw. or work$ status.tw. or work$ situation$.tw. or exp occupation/ or occupation$.tw. or exp soldier/ or exp fire fighter/ or exp agricultural worker/ or exp construction worker/ or exp army/ or agricultur$.mp. or Military Personnel.mp. or military.mp. or construction.mp. or occupation$.tw. or occupational activit$.tw. or occupational exposure$.tw. | 1,821,601 |
| 8 | exp total hip arthroplasty/ or exp hip replacement/ or exp hip arthroplasty/ or exp hip prosthesis/ | 65,486 |
| 9 | exp prosthesis failure/ or exp failure free survival/ or exp kaplan meier method/ or exp survival rate/ or exp survival/ or exp reoperation/ or (prosthesis failure or joint$ fail$ or survival rate$ or survivorship or survival analy$ or reoperation or revision).mp. | 1,546,626 |
| 10 | 1 or 2 or 5 or 8 | 118,948 |
| 11 | 4 and 9 and 10 | 2,631 |
| 12 | 7 and 9 and 10 | 692 |
| 13 | 3 and 9 and 10 | 5,164 |
| 14 | 3 and 7 and 10 | 267 |
| 15 | 6 and 10 | 145 |
| 16 | exp exercise/ or exp sport/ or exp physical activity/ or (exercise or sport$ or physical activit$).mp. | 1,048,406 |
| 17 | 9 and 10 and 16 | 2,770 |
| 18 | 11 or 12 or 13 or 14 or 15 or 17 | 10,374 |
| 19 | limit 18 to ((conference abstracts or embase) and (english or spanish) and yr="1985 -Current") | 8,383 |
| 20 | limit 19 to human | 7,975 |

**Ovid MEDLINE(R) ALL** 1946 to June Week 5 2021

|  | **Query** | **Results from 8 Jul 2021** |
| --- | --- | --- |
| 1 | exp Arthroplasty, Replacement, Knee/ or exp knee prosthesis/ | 32,298 |
| 2 | (total knee replacement$ or knee replacement$ or knee arthroplast$ or gonarthroplasty or TKA or knee prosthesis).mp. | 33,425 |
| 3 | exp Arthroplasty, Replacement, Hip/ or exp Hip Prosthesis/ | 43,013 |
| 4 | (hip prosthesis or hip replacement$ or total hip replacement$ or total hip arthroplast$ or THA).mp. | 42,545 |
| 5 | 1 or 2 or 3 or 4 | 77,804 |
| 6 | exp Survival/ or exp Survival Rate/ or exp Survival Analysis/ or exp Disease-Free Survival/ or exp Kaplan-Meier Estimate/ or exp Prosthesis Failure/ or exp Prosthesis-Related Infections/ | 505,443 |
| 7 | (survivorship or survival$ analy$ or kaplan meier or Survival Rate$ or reoperation or re-operation or (prosthesis fail$ or joint$ fail$)).mp. | 609,533 |
| 8 | 6 or 7 | 695,011 |
| 9 | exp Occupations/ or exp Employment/ or exp Work/ or exp Farmers/ or exp Military Personnel/ or exp Miners/ or exp Firefighters/ or exp Agricultural Worker/ or exp soldier/ | 212,583 |
| 10 | (occupational exposure$ or (manual labor or manual labour) or (employment or employment status)).mp. or work.ab,ti. or work activit$.ab,ti. or work$ status.ab,ti. or work$ situation$.ab,ti. or occupation$.ab,ti. or occupational activit$.mp. or Soldiers.mp. or Agricultural Workers.mp. or Construction worker.mp. or Army.mp. or agricultur$.mp. or Military Personnel.mp. or military.mp. or Painter construction.mp. or Bricklayer construction.mp. or Stonemason construction.mp. or Construction carpenter.mp. or Construction joiner.mp. | 1,223,404 |
| 11 | 9 or 10 | 1,293,046 |
| 12 | exp "Return to Work"/ or return$ to work.mp. or work resumption.mp. or back to work.mp. | 12,061 |
| 13 | (long term or long-term or longterm).mp. | 759,672 |
| 14 | exp Risk Factors/ or risk factor.mp. | 970,369 |
| 15 | exp Sports/ or exp Exercise/ or activities, physical.mp. or activity, physical.mp. or exercise, physical.mp. or exercise training.mp. or physical activities.mp. or physical activity.mp. | 362,492 |
| 16 | 5 and 8 and 14 | 2,460 |
| 17 | 5 and 8 and 11 | 424 |
| 18 | 5 and 8 and 13 | 3,543 |
| 19 | 5 and 11 and 13 | 164 |
| 20 | 5 and 12 | 120 |
| 21 | 5 and 8 and 15 | 306 |
| 22 | 16 or 17 or 18 or 19 or 20 or 21 | 6,435 |
| 23 | limit 22 to (humans and yr="1985 -Current" and (english or spanish)) | 5,734 |

**Scopus**

TITLE-ABS-KEY ( ( "knee prosthesis"  OR  "tka"  OR  "gonarthroplasty"  OR  "knee arthroplast*"  OR  "knee replacement*"  OR  "total knee replacement*"  OR  "total hip replacement*"  OR  "hip replacement"  OR  "total hip arthroplast*"  OR  "THA"  OR  "hip prosthesis" )  AND  ( "Survivorship"  OR  " survival* analy*"  OR  "Kaplan Meier"  OR  "survival rate*"  OR  "reoperation"  OR  "prosthesis fail*"  OR  "joint* fail*" )  AND  ( "risk factor*"  OR  "long-term"  OR  "long term"  OR  "longterm"  OR  "employment"  OR  "employment status"  OR  "work"  OR  "work activit*"  OR  "work* status"  OR  "work* situation"  OR  "occupation*"  OR  "occupational activit*"  OR  "occupational exposure*"  OR  "manual labour"  OR  "weight lifting"  OR  "squatting"  OR  "kneeling"  OR  "military personnel"  OR  "farmer*"  OR  "miners"  OR  "agriculture work*"  OR  exercise  OR  sport*  OR  "physical activit*"  OR  "winter sport*"  OR  "racquet sport*"  OR  "running" ) )  AND  PUBYEAR  >  1985  AND  ( LIMIT-TO ( DOCTYPE ,  "ar" )  OR  LIMIT-TO ( DOCTYPE ,  "re" )  OR  LIMIT-TO ( DOCTYPE ,  "cp" ) )  AND  ( LIMIT-TO ( LANGUAGE ,  "English" )  OR  LIMIT-TO ( LANGUAGE ,  "Spanish" ) )
