# Supplementary material for: Risk of revision arthroplasty surgery after exposure to physically demanding occupational or leisure activities: A systematic review
Source: PLoS One. 2022 Feb 28;17(2):e0264487. doi: 10.1371/journal.pone.0264487 (PMC8884506; doi:10.1371/journal.pone.0264487)
Supplement: S1 Table — (DOCX) [file pone.0264487.s002.docx]

**S1 Table. Quality assessment of cohort studies retrieved**

| **ITEMS ^a^** | **Dubs et al** | **Kilgus et al** | **Inoue et al** | **Maurer et al** | **FLugsrud et al** | **Lübbeke et al** | **Ollivier et al** | **Heck et al** | **Crawford et al** | **Ponzio et al** |
| --- | --- | --- | --- | --- | --- | --- | --- | --- | --- | --- |
| 1. The study adressess an appropiate and clearly focused question | CS | Y | Y | Y | Y | Y | Y | N | Y | Y |
| 1. Are the exclusion and inclusion criteria clearly reported? | Y | N | Y | Y | Y | Y | Y | N | Y | Y |
| 3. The two groups being studied are selected from the source populations that are comparable in all respects other than the factor under investigation | CS | Y | Y | Y | Y | Y | Y | N | CS | Y |
| 4. The study indicates how many of the people asked to take part did so, in each of the groups being studied | CS | N | CS | CS | NA | N | N | N | CS | CS |
| 5. What percentage of individuals or clusters recruited into each arm of the study dropped out before the study was completed | NA | NA | CS | NA | CS | Y | CS | CS | CS | NA |
| 7. Comparison is made between full participants and those lost to follow up, by exposure status | CS | N | N | N | N | N | CS | N | N | N |
| 8. The outcomes are clearly defined | N | Y | Y | Y | Y | Y | Y | Y | Y | Y |
| 9. The assessment of outcome is made blind to exposure status. If the study is retrospective this may not be applicable | NA | NA | CS | NA | Y | Y | NA | N | NA | NA |
| 10. Where blinding was not possible, there is some recognition that knowledge of exposure status could have influenced the assessment of the outcome | NA | CS | CS | NA | NA | NA | NA | N | NA | NA |
| 11. The method of the assessment of exposure is reliable | N | CS | N | CS | Y | Y | Y | N | Y | Y |
| 12. Evidence from another sources is used to demonstrate that the method of outcome assessment is valid and reliable | N | CS | N | NA | Y | Y | N | N | NA | N |
| 13. Exposure level or prognostic factors is assessed more than once | N | N | N | N | N | Y | N | N | CS | N |
| 14, Is the information regarding the patients who did not gave informed consent and who were not willing to participate adequately reported? | NA | N | N | N | NA | N | N | N | N | CS |
| 15. Are the baseline characteristics of included patients reported? | N | Y | N | Y | N | Y | Y | N | Y | Y |
| 16. Is the surgical technique adequately reported? | N | N | N | Y | N | Y | N | N | Y | N |
| 17. Are the prosthesis brand and fixation adequately reported? | N | Y | Y | Y | N | Y | Y | N | Y | N |
| 18. Are the reasons or definition for revision adequately reported? | N | Y | NA | Y | Y | Y | Y | Y | Y | Y |
| 19. Are the number of revisions or revisions rates regarding aseptic loosening (either KM or life table of revisions per 100 observed component years) adequately reported? | Y | Y | N | Y | Y | N | Y | N | Y | Y |
| 20. How were the cohorts constructed? (Consecutively / non-consecutively / unknown / not applicable) ^b^ | CS | CS | Y | Y | NA | Y | CS | CS | Y | CS |
| 21. How adequate was the follow-up? | CS | CS | CS | Y | Y | Y | NA | N | Y | CS |
| 22. How was the follow-up performed? (predefined / non-predefined/ unknown / not applicable) ^c^ | CS | N | CS | Y | NA | Y | CS | N | CS | CS |
| 23. How many arthroplasties are at risk at the follow-up of interest? (≥ 20 / <20 / unknown) ^d^ | Y | Y | Y | Y | Y | Y | Y | N | Y | Y |
| 24. The main potential confounders are identified and taken into account in the design and analysis? | CS | N | Y | Y | Y | NA | Y | N | Y | Y |
| 25. Have confidence intervals been provided? | CS | N | Y | Y | Y | N | Y | N | Y | Y |
| 26. Has a worse case analysis or competing risks analysis for competing endpoints been performed? | CS | N | N | Y | N | N | N | N | N | N |
| **Quality assessment ^e^** | - | - | 0 | + | + | + | + | - | + | + |
| **Risk of bias** | H | H | H | M | M | L | M | H | M | M |

^a^ Items 1, 3-13, 24 and 25 are from the Scottish Intercollegiate Guidelines Network (SIGN) checklist, and items 2, 14-23 and 26 are from the Assessment of Quality in Lower Limb Arthroplasty (AQUILA) methodological quality assessment checklist

^b^ Yes if consecutively, no if non-consecutively, can’t say if unknown or not applicable

^c^ Yes if predefined, no if non-predefined, can’t say if unknown or not applicable

^d^ Yes if number of arthroplasties ≥20, no if number of arthroplasties <20 and can’t say if unknown.

^e^ Quality assessed as: high ++, acceptable +, poor 0, very poor –

Y, yes; N, no; CS can’t say; NA, not applicable; H, high; M, moderate and L, low
