# Supplementary material for: Risk of revision arthroplasty surgery after exposure to physically demanding occupational or leisure activities: A systematic review
Source: PLoS One. 2022 Feb 28;17(2):e0264487. doi: 10.1371/journal.pone.0264487 (PMC8884506; doi:10.1371/journal.pone.0264487)
Supplement: S2 Table — (DOCX) [file pone.0264487.s003.docx]

**S2 Table. Quality assessment of case-control studies retrieved**

| **ITEMS ^a^** | **Espehaug et al ^c^** | **Delfin et al** | **Jones et al** |
| --- | --- | --- | --- |
| 1. The study addresses an appropriate and clearly focused question | Y | Y | Y |
| 2. The cases and controls are taken from comparable populations | Y | CS | Y |
| 3. The same exclusion criteria are used for both cases and controls | CS | Y | Y |
| 4. What percentage of each group (cases and controls) participated in the study? | N | Y | Y |
| 5. Comparison is made between participants and non-participants to establish their similarities or differences | Y | N | Y |
| 6. Cases are clearly defined and differentiated from controls | Y | Y | Y |
| 7. It is clearly established that controls are non-cases | Y | Y | Y |
| 8. For matched studies, give matching criteria and the number of controls per case | Y | Y | Y |
| 9. Measures will have been taken to prevent knowledge of primary exposure influencing case ascertainment | NA | Y | CS |
| 10. Exposure status is measured in a standard, valid and reliable way | Y | Y | Y |
| 11. Report number of individuals at each stage of the study | N | Y | Y |
| 12. The main potential confounders are identified and taken into account in the design and analysis | Y | CS | Y |
| 13. Describe all statistical methods, including those used to control for confounding | Y | Y | Y |
| 14. Explain how missing data are addressed | N | CS | CS |
| 15. Confidence intervals are provided | Y | Y | Y |
| 16. How well was the study done to minimize the risk of bias or confounding? | Y | Y | Y |
| 17. Discuss both direction and magnitude of any of the potential bias | Y | Y | Y |
| **Quality of assessment ^b^** | + | ++ | ++ |
| **Risk of bias** | L | L | L |

^a^ Items 1-7, 9, 10, 12 and 15-16 are from the Scottish Intercollegiate Guidelines Network (SIGN) checklist, and items 8, 11, 13-14, 17 are items from the Strengthening the Reporting of Observational Studies in Epidemiology (STROBE) list.

^b^ Quality assessed as: high ++, acceptable +, poor 0, very poor –

^c^ Quality of assessment and risk of bias rated separately for LTPA (+/Low) and occupation (0 / Moderate) due to differences in the accuracy of exposure reported

Y, yes; N, no; CS can’t say; NA, not applicable; H, high; M, moderate and L, low
