# Supplementary material for: Risk of revision arthroplasty surgery after exposure to physically demanding occupational or leisure activities: A systematic review
Source: PLoS One. 2022 Feb 28;17(2):e0264487. doi: 10.1371/journal.pone.0264487 (PMC8884506; doi:10.1371/journal.pone.0264487)
Supplement: Attachment — Submitted filename: 211126 Reviewer comments.docx [file pone.0264487.s004.docx]

Thank you for allowing me to review your interesting manuscript on the evidence for risk of revision arthroplasty related to physically demanding occupation and leisure time activities

**General remark**: Please carefully check the manuscript for grammatical errors and incorrect sentencing once more

**Title:** no comments

**Abstract**: introduction is not logical to me, please make it more clear to the reader how you get from primary osteoarthritis to revision arthroplasty in this intro
Add July to ‘2021’ for your search.

The conclusion of your abstract does not state a conclusion based on your results (effect of PA on revision risk).

**Introduction**: well written
line 77: your reference only supports this statement for knee arthroplasty patients

**Material and methods**:
Line 130 screening for title and abstract by one author, with only a random 10% checked by two other authors, limits the validity of this first screening step in my opinion.

Information on grading of physically-demanding activities (low, intermediate, high) is lacking in the methods section. How did the authors define this?

**Results**:

Overall, the results section is too long.
Suggest adding your quality assessment as supplementary material. Did you do anything with the quality assessment, i.e. did you sort your results based on the quality of the articles?
Line 179-180 incomplete sentence
Line 190: 5,328 is misleading because the authors only included 1,008 in each group for their analysis.
Line 193: I’m surprised by the prevalence of >60%, I was under the impression that you only included primary OA cases?
Line 266-268: discussion remark
Line 283-285: discussion remark
Line 315-316: discussion remark
Table 4: last column has ‘Risk estimate 95% CI’ as a header but most studies did not report a 95% CI so this header is not accurate here.
Line 333: did the authors also combine all reasons for revision in the low- and high-activity cohorts? Would be more interesting than the separate analyses for each reason for revision.

**Discussion:**
Could be shorter but I enjoyed reading it.
Line 378 and throughout the manuscript ‘as to whether’ is used excessively
Line 382: these numbers don’t necessarily suggest that the topic is understudied; maybe the 13 existing studies sufficiently answered the question?

**Conclusion**:
Please provide the reader with a general summary of your own results, not just the statement that limited evidence is available (they know by now and this is not the most important finding you want them to remember, I guess?). “In conclusion, based on limited evidence, occupational PA and LTPA do not convincingly increase the risk of revision hip and knee arthroplasty in primary arthroplasty patients” or something like that.

**References**
No comments
